# Einfluss von Dysphagie- und ernährungsabhängigen Faktoren auf das Überleben von Patienten mit Larynx- und Hypopharynxkarzinomen: Eine retrospektive Kohortenstudie

**DOI:** 10.1007/s00106-025-01706-0

**Published:** 2026-01-12

**Authors:** Sarah Vester, Julian Künzel, Sophia Grabner, Cosima Dillinger, Michael Gerken, Stephan Dürr, Christopher Bohr, Peter Kummer

**Affiliations:** 1 Klinik und Poliklinik für Hals-Nasen-Ohrenheilkunde, Abteilung Phoniatrie und Pädaudiologie, Universitätsklinikum Regensburg, Regensburg, Deutschland; 2 Klinik und Poliklinik für Hals-Nasen-Ohrenheilkunde, Universitätsklinikum Regensburg, Regensburg, Deutschland; 3 Tumor Zentrum Regensburg, Zentrum für Qualitätssicherung und Versorgungsforschung, Fakultät für Medizin, Universität Regensburg, Regensburg, Deutschland; 4 Universitätsklinikum Regensburg, Franz-Josef-Strauss Allee 11, 93053 Regensburg, Deutschland

**Keywords:** Kopf-Hals Tumoren, Schluckstörungen, Mangelernährung, Pneumonie, Gesamtüberleben, Head and neck neoplasms, Deglutition disorders, Malnutrition, Pneumonia, Overall survival

## Abstract

**Hintergrund und Fragestellung:**

Neben dem tumorbedingten Überleben werden bei Kopf-Hals-Tumoren aufgrund von Lokalisation und onkologischer Therapie wesentliche, nichttumorbedingte, interventionsfähige Risiken zunehmend erkannt. Vor allem Schluckstörungen haben einen Einfluss auf das Gesamtüberleben, besonders aufgrund von Folgen wie Aspirationspneumonien und Mangelernährung. Die vorliegende Arbeit zielte darauf ab, an einem typischen Patientenkollektiv eines deutschen Kopf-Hals-Tumorzentrums diese Risiken und mögliche Einflussfaktoren zu erfassen.

**Methoden:**

Im Rahmen einer retrospektiven Kohortenstudie wurden Patienten mit Hypopharynx- und Larynxkarzinomen untersucht. Hierbei wurde mittels uni- und multivariabler logistischer Regression der Einfluss von unabhängigen Variablen auf dysphagie- und ernährungsabhängige Outcome-Variablen untersucht sowie anhand von Kaplan-Meier-Kurven und der Cox-Regressionsanalyse Einflussfaktoren auf das Überleben ermittelt.

**Ergebnisse:**

Für 214 Patienten wurden prätherapeutisches Untergewicht (Hazard Ratio, HR: 2,025) und Pneumonie (HR 1,569) als signifikante Risikofaktoren für das Überleben identifiziert. Gleichzeitig wurde prätherapeutisches Untergewicht als Einflussfaktor für die prätherapeutische Dysphagie (OR 7,6) nachgewiesen. Diese Faktoren stellen ihrerseits einen Einflussfaktor auf die posttherapeutische Dysphagie dar (Odds Ratio, OR: 12,1). Das Vorliegen einer prä- und posttherapeutischen Dysphagie hatte einen signifikanten Einfluss (OR 4,0) auf die Entstehung von Pneumonien.

**Schlussfolgerung:**

Die Studienergebnisse weisen darauf hin, dass eine frühzeitige und konsequente Therapie der Mangelernährung und Dysphagie das Langzeitüberleben verbessern könnte. Weitere Studien zu Interventionen dieser Einfluss- und Risikofaktoren sind notwendig, um die Outcomebeurteilung prä-, früh- und stationärer Rehabilitationsmaßnahmen vor, während bzw. nach onkologischer Therapie zu prüfen. Diese Untersuchungen sollten jenseits allein subjektiver, patientenzentrierter Erfolgsmessung, auf die mit Dysphagie verbundenen Folgen der Krankheitsbewältigung, Lebensqualität, Morbidität und Mortalität, sowie der mit ihren Komplikationen verbundenen Kosten erweitert werden.

## Abstract (en)

**Background and objective:**

In addition to cancer-related survival, significant non-cancer-related modifiable risk factors are increasingly recognized in head and neck cancer due to disease localization and oncological therapy. Dysphagia in particular has an impact on overall survival, especially due to consequences such as aspiration pneumonia and malnutrition. This study aimed to assess these risks and possible influencing factors in a typical patient population of a German head and neck cancer center.

**Methods:**

Patients with hypopharyngeal and laryngeal cancer were examined as part of a retrospective cohort study. Uni- and multivariable logistic regression was used to investigate the influence of independent variables on dysphagia- and diet-dependent outcome variables. Factors influencing survival were identified using Kaplan–Meier curves and Cox regression analysis.

**Results:**

Pretherapeutic underweight (hazard ratio, HR, 2.025) and pneumonia (HR 1.569) were identified as significant risk factors for survival in 214 patients. At the same time, pretherapeutic underweight was found to be an influencing factor for pretherapeutic dysphagia (OR 7.6), which, in turn, was an influencing factor for posttherapeutic dysphagia (odds ratio, OR, 12.1). The presence of pre- and posttherapeutic dysphagia had a significant influence (OR 4.0) on the development of pneumonia.

**Conclusion:**

The results indicate that early and consistent treatment of malnutrition and dysphagia could improve long-term survival. Further studies on interventions addressing these influencing and risk factors are necessary to extend the outcome evaluation of pretreatment, early, and inpatient rehabilitation measures after oncological therapy beyond solely subjective patient-centered outcome measures. These future investigations should also address the consequences of dysphagia on disease management, quality of life, morbidity, and mortality as well as the costs associated with its complications.

## Epidemiologie

Weltweit zählen Kopf-Hals-Tumoren, einschließlich Lippen‑, Mundhöhlen‑, Pharynx- und Larynxkarzinomen zu den siebthäufigsten Krebserkrankungen. Jährlich werden etwa 660.000 neue Fälle und 325.000 Todesfälle gemeldet [11].

In Deutschland erkrankten in den Jahren 2016 und 2018 im Jahr durchschnittlich 13.449 Männer und 5336 Frauen an Kopf-Hals-Tumoren, das entspricht einer altersstandardisierten Inzidenz von 23,7 Männern und 8,4 Frauen pro 100.000 Einwohner. Der mediane Altersdurchschnitt bei Erstdiagnose lag bei 65 bzw. 66 Jahren [12].

Die Prävalenz einer Dysphagie liegt für Kopf-Hals-Tumorpatienten abhängig von der Tumorgröße und Lokalisation bei bis zu 80 % [15, 22]. Komplikationen der Dysphagie sind neben Aspirationspneumonien mit einer erhöhten Mortalität auch soziale Isolation und Verlust der Lebensqualität [19, 21]. Bei 30 % der Patienten besteht bereits vor Beginn der Therapie eine Malnutrition, weitere 30 % zeigen Anzeichen einer drohenden Mangelernährung [1], und diese wird durch Komplikationen der Therapie, wie z. B. Dysphagie, Dysgeusie und Fatigue, zusätzlich verstärkt [5].

Ausgehend von diesen Zahlen stellt sich die Frage, welchen Einfluss Dysphagie und dysphagieabhängige Faktoren (z. B. Aspiration, Malnutrition sowie ernährungsabhängige Faktoren wie Gewichtsverlauf, Body-Mass-Index [BMI]) auf das Überleben von Kopf-Hals-Tumorpatienten haben. Um diese Fragestellung weiter zu beleuchten, wurden Daten zu Hypopharynx- und Larynxkarzinomen ausgewertet und Einflussfaktoren auf das Überleben herausgearbeitet.

## Material und Methoden

Es wurde eine retrospektive Kohortenstudie aus Daten des klinischen Krebsregisters Ostbayern in Kooperation mit dem Zentrum für Qualitätssicherung und Versorgungsforschung der Universität Regensburg durchgeführt. Für die Studie liegt ein positives Votum der Ethikkommission der Universität Regensburg (Nr.: 20-2055-104) vor.

### Auswahl geeigneter Variablen

Prognostisch relevante Variablen und potenziell prädiktive Faktoren wurden anhand der klinischen Erfahrung und einer Literaturrecherche ermittelt [4, 7, 16]. Getrennt nach patientenbezogenen Variablen (z. B. Alter, Geschlecht, Performance-Status der Eastern Cooperative Oncology Group [ECOG]), tumorbezogenen Variablen (z. B. Tumorstadium, Lokalisation, Therapie), ernährungsabhängigen Variablen (z. B. Gewicht, BMI, Versorgung mit perkutaner endoskopischer Gastrostomie [PEG]) und dysphagieabhängigen Variablen (z. B. Vorhandensein einer Dysphagie, Auftreten von Pneumonien) wurde eine umfangreiche Variablenliste erstellt.

### Datensatz

Der initiale Datensatz umfasste alle gemeldeten Erstdiagnosen eines Kopf-Hals-Tumors in den Jahren 2012–2017 im Zuständigkeitsbereich des Comprehensive Cancer Center Ostbayern (CCCO). Hieraus ergibt sich bis zum Zeitpunkt der Datenauswertung 2022 eine minimale Nachbeobachtungszeit von 5 Jahren.

### Datenerhebung und -verwaltung

Die Tab. 1 zeigt die angewendeten Ein- und Ausschlusskriterien. Kleine Stimmlippenkarzinome wurden aufgrund der klinisch sehr selten vorhandenen Schlucksymptomatik zur Vermeidung eines Selektionsbias ausgeschlossen.

**Tab. 1:** Ein- und Ausschlusskriterien

Einschlusskriterien	Ausschlusskriterien
Alter > 18 Jahre	Stimmlippenkarzinome des Stadiums T0–2
Erstdiagnose eines Hypopharynx oder Larynxkarzinoms (nach ICD-10)	
Stationäre Diagnostik oder Behandlung des Tumors am UKR	
Erstdiagnose zwischen 01.2012 und 12.2017	
Invasives Plattenepithelkarzinom	
Vorhandenes UICC-Stadium	

*ICD *International Classification of Diseases*, UKR *Universitätsklinikum Regensburg*, UICC *Union Internationale Contre le Cancer

Die Variablen wurden mithilfe des Klinikverwaltungsprogramms SAP (SAP Deutschland SE & Co. KG, Walldorf, Deutschland) und der Patientenakte erhoben, teilweise handelt es sich um anamnestische Angaben oder Diagnosen nach Funktionsuntersuchungen, wie beispielsweise Schluckendoskopien.

Die Patientendaten wurden pseudonymisiert in Excel (Version 2018, Microsoft Corp., Redmond/WA, USA) gesammelt und zur Auswertung in SPSS Statistics (Version 29, IBM, Armonk/NY, USA) übertragen.

Der initiale Datensatz wurde zunächst auf das Vorliegen der Ein- und Ausschlusskriterien geprüft und deskriptiv analysiert. Für die multivariable statistische Analyse wurde ein bereinigter Datensatz angefertigt, hierfür mussten weitere Fälle mit unzureichender Datenlage ausgeschlossen werden.

Um die Unklarheit in Bezug auf die Überlebensdaten der Patienten zu minimieren, erfolgte im Oktober 2022 eine „Lebend-Abfrage“ über die Einwohnermeldeämter und eine Aktualisierung der Sterbedaten.

### Statistik

Nach initial deskriptiver Analyse wurden für die weitere Auswertung essenzielle Zielvariablen festgelegt: prätherapeutisches Untergewicht, prä- und posttherapeutische Dysphagie und eine stattgehabte Pneumonie während und nach der Therapie.

Im ersten Schritt wurden diese mithilfe von Kreuztabellen und dem Chi-Quadrat-Test nach Pearson auf signifikante Korrelationen zu möglichen Einflussvariablen untersucht. Ein Signifikanzniveau von 0,05 wurde definiert. Der Zusammenhang zwischen Ziel- und Einflussvariablen wurde untersucht und als Odds Ratio (OR) dargestellt, univariabel und anschließend in einem multivariablen Modell, um eine Hierarchie der Einflussfaktoren in einem zueinander adjustierten Gesamtmodell herauszuarbeiten.

Im dritten Schritt erfolgte eine Überlebenszeitanalyse mit Kaplan-Meier-Kurven, durch das letzte Lebenddatum ist die Überlebenszeitanalyse zensiert und bezieht sich ausschließlich auf die Patienten, die nachweislich verstorben sind. Zur Verifizierung statistisch signifikanter Differenzen in den Vergleichsgruppen wurde der Log-Rank-Test angewandt.

Zuletzt wurden uni- und multivariable Cox-Proportional-Hazard-Modelle angewendet, wobei zu Beginn der prognostischen Risikoanalysen univariable Regressionen ohne Einflussnahme anderer Faktoren durchgeführt wurden. Dies diente dazu, Kolinearitäten und mögliche Confounder und Moderator- bzw. Mediatorvariablen zu identifizieren. Um die wichtigsten Faktoren gegeneinander adjustieren zu können, wurden nachfolgend multivariable Regressionsmodelle mit unterschiedlichen Faktorenkonstellationen berechnet.

## Ergebnisse

### Patientenkollektiv und Therapie

Aus dem initialen Datensatz konnten 231 Fälle mit Erstdiagnose Hypopharynx- und Larynxkarzinom identifiziert werden. Nach Prüfung aller Ein- und Ausschlusskriterien verblieben 222 Patienten zur deskriptiven Analyse, davon 41,4 % (*n* = 92) mit Larynxkarzinom und 58,6 % (*n* = 130) mit Hypopharynxkarzinom.

Das Kollektiv bestand zu 90 % (*n* = 200) aus Männern und zu 10 % (*n* = 22) aus Frauen, das Durchschnittsalter lag bei 64 Jahren, wobei Männer im Schnitt etwas früher erkrankten als Frauen.

Vor Therapie gaben insgesamt 88,3 % (*n* = 196) der Patienten Nikotinkonsum und 78,8 % (*n* = 175) regelmäßigen Alkoholkonsum an. Nach Therapie verzichteten 35 % (*n* = 78) darauf.

Mit Werten von ECOG 0 und 1 zeigten 61,7 % der Patienten einen guten Performance-Status, aufgrund des altersadjustieren Charlson-Komorbiditätsindex (Charlson Comorbidity Index, CCI) war bei 80,6 % der Patienten hingegen eine Einjahresmortalitätsrate von 85 % zu schätzen.

Bei 75,2 % der Patienten wurde ein fortgeschrittenes T‑Stadium (T3 und T4) mit Lymphknotenbefall (73,9 %) diagnostiziert, bei 8,6 % Fernmetastasen.

Die Therapie bestand in 9,0 % der Fälle aus alleiniger Operation, in 24,8 % aus primärer Operation mit adjuvanter Therapie und in 48,6 % aus primärer Radiatio oder Radiochemotherapie. In 17,6 % der Fälle fand eine palliative Therapie statt, oder es fehlten die Daten zu Therapie.

### Ernährungsabhängige und schluckspezifische Variablen

Als ernährungsabhängige Variablen wurden der BMI, dessen Entwicklung im Verlauf und das Vorhandensein einer PEG-Sonde erhoben. PEG-Sonden werden in unserer Klinik bei bestehenden oder durch die Therapie zu erwartenden schweren Schluckstörungen gelegt.

**Abb. 1:**
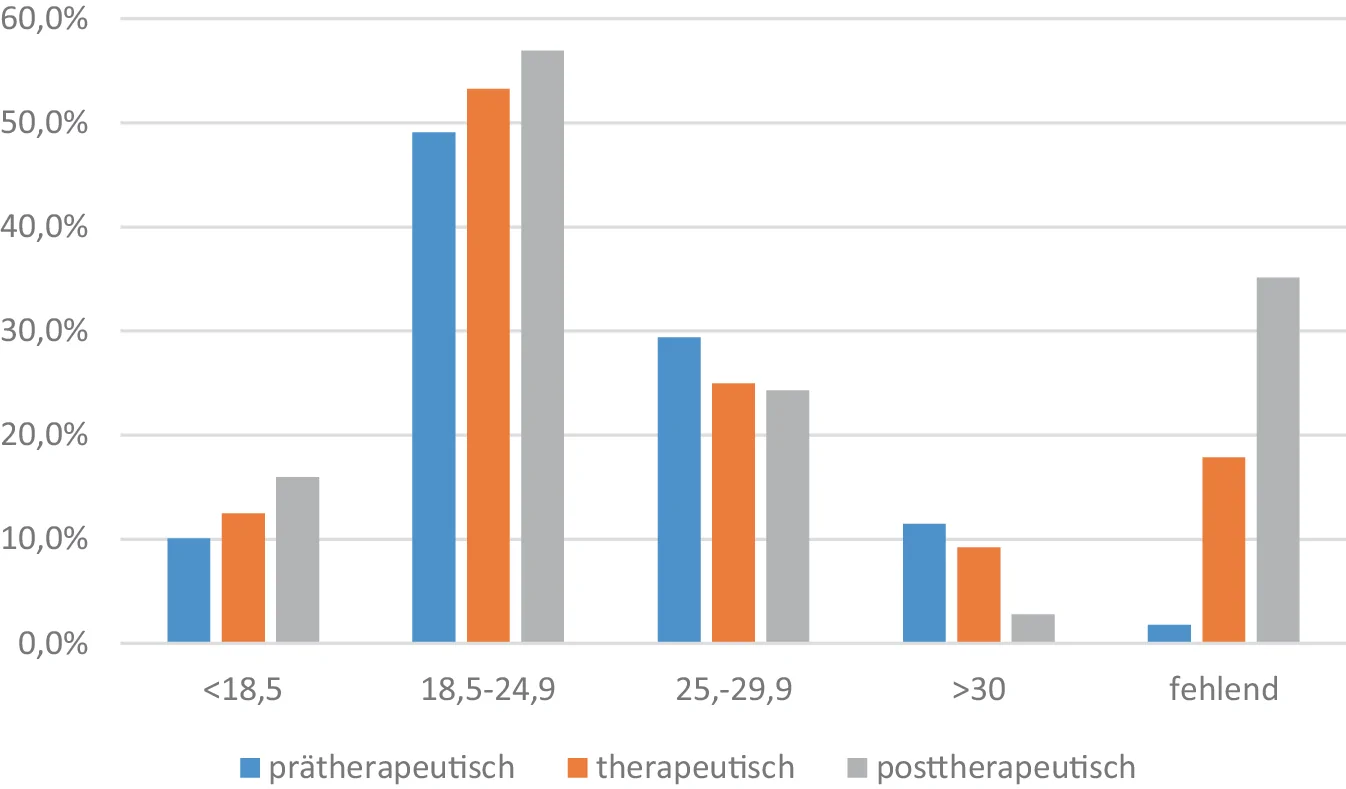
Verteilung und Entwicklung des Body-Mass-Index (BMI; kg/m^2^) im Verlauf

Die Abb. 1 zeigt die Verteilung und den zeitlichen Verlauf des BMI. Soweit im Therapieverlauf trotz zunehmend fehlender Werte erkennbar, kommt es zu einer Abnahme übergewichtiger und Zunahme mangelernährter Patienten.

Im Rahmen der Tumorbehandlung wurden fast 3/4 aller Patienten (175/222) via PEG ernährt, 3–6 Monate nach Therapieende noch 15,8 % vollständig und 22,9 % teilweise .

Vor Beginn der Therapie litten bereits 55 % (*n* = 122) der Patienten an einer Dysphagie, nach Abschluss der Therapie 63,5 % (*n* = 141), von diesen 141 Patienten hatten 6,4 % eine Operation, 24,3 % eine Operation mit adjuvanter Radiatio/Radiochemotherapie und 57,9 % eine primäre Radiatio/Radiochemotherapie.

Eine Pneumonie entwickelte sich bei 60 Patienten, 25 % davon vor der Therapie, 30 % währenddessen und 41,7 % danach; bei 2 Patienten fehlten Angaben zum Zeitpunkt. 11,4 % der Pneumoniepatienten hatten eine alleinige Operation, 20,5 % eine Operation mit adjuvanter Therapie und 59,1 % eine Radiatio/Radiochemotherapie.

Eine stationäre Rehabilitation erhielten im Anschluss an die Therapie 34,3 % aller Patienten, mehr als die Hälfte (56,7 %) nahm keine Rehabilitation in Anspruch. Nur die Hälfte der Patienten (46,5 %) mit einer posttherapeutischen Dysphagie nahm eine Rehabilitationsmaßnahme wahr, tendenziell waren diese Patienten jünger, ein statistisch signifikanter Zusammenhang zum Alter bestand, wie auch für den ECOG-Status, nicht.

### Prognosemodelle

Für ein Prognosemodell der dysphagie- und ernährungsabhängigen Variablen, d. h. der Zielvariablen prätherapeutisches Untergewicht, prä- und posttherapeutische Dysphagie und Pneumonie, wurden die Daten von 214 Patienten verwendet, bereinigt um 8 Patienten mit lückenhaftem Datensatz. Die Tab. 2 zeigt Zusammenhänge zwischen Einflussvariablen nach entsprechender Vorprüfung in Bezug auf die hier betrachteten Zielvariablen.

**Tab. 2:** Univariable und multivariable Analysen schluck- und ernährungsbezogener Zielvariablen

Abhängige Zielvariablen	Untergewicht vor Therapiebeginn (BMI < 18,5)		Dysphagie vor Therapiebeginn		Dysphagie nach Therapie		Pneumonie	
*Unabhängige Einflussvariablen*	Univariable OR 95 %-KI	Multivariable OR 95 %-KI	Univariable OR 95 %-KI	Multivariable OR 95 %-KI	Univariable OR 95 %-KI	Multivariable OR 95 %-KI	Univariable OR 95 %-KI	Multivariable OR 95 %-KI
*Geschlecht w. vs. m.*^*+*^	0,405 0,052–3,172	0,442 0,037–5,302	1,279 0,507–2,073	1,214 0,345–4,275	0,423 0,167–1,074	0,288 0,068–1,216	0,618 0,174–2,200	0,635 0,105–3,853
*Alterskategorie (Jahre)*								
60–69 vs. < 60^+^	0,662 0,145–2,563	0,609 0,145–2,563	1,089 0,572–2,073	1,606 0,686–3,762	0,949 0,472–1,909	0,763 0,282–2,062	0,492 0,205–1,179	0,509 0,168–1,539
> 69 vs. < 60^+^	1,536 0,555–4,248	1,403 0,281–7,005	0,944 0,485–1,841	1,744 0,627–4,856	0,912 0,440–1,892	0,940 0,285–2,062	1,301 0,605–2,799	2,212 0,677–7,230
*Noxenkonsum prätherapeutisch*								
Zigarettenkonsum vs. keine Noxen^+^	1,050 0,155–7,127	0,14 0,007–2,940	0,467 0,129–1,692	0,439 0,077–2,508	1,625 0,435–6,068	0,643 0,079–5,257	1,842 0,311–10,915	5,864 0,421–81,732
Alkoholkonsum vs. keine Noxen^+^	–	0,00	1,556 0,218–11,086	0,271 0,006–11,559	–	–	–	2,014
Beide Noxen vs. keine Noxen^+^	0,757 0,158–3,633	0,226 0,20–2,609	1,167 0,414–3,286	1,502 0,319–7,067	2,404 0,852–6,784	0,368 0,051–2,661	1,953 0,424–8,994	5,178 0,428,62,670
**ECOG 3–4 vs. 0–2**^**+**^	4,444^*^ **1,212–16,302**	10,832^*^ **1,397–83,997**	2,113 0,635–3,286	3,059 0,412–22,683	1,600 0,320–7,990	0,412 0,031–5,468	0,667 0,142–3,134	0,0
*CCI*								
3**–**4 vs. 1**–**2^+^	–	–	0,815 0,349–1,900	0,229^*^ **0,071–0,737**	1,547 0,646–3,705	2,128 0,533–8,503	1,909 0,525–6,943	1,595 0,324–7,840
≥ 5 vs. 1**–**2^+^	–	–	1,511 0,620–3,683	0,551 0,164–1,853	1,625 0,651–4,059	1,011 0,241.4,245	2,759 0,749–10,157	2,460 0,485–12,489
*BMI prätherapeutisch*								
< 18,5 vs. 18,5–24,9^+^	–	–	4,330^*^ **1,372–13,670**	7,558^*^ **1,394–40,981**	2,148 0,582–7,932	1,668 0,166–16,811	1,185 0,419–3,354	0,847 0,201–3,568
25–29,9 vs. 18,5–24,9^+^	–	–	1,424 0,753–2,692	2,038 0,895–4,640	0,542 0,279–1,055	0,228^**^ **0,084–0,623**	0,608 0,270–1,370	0,379 0,118–1,219
30 vs. 18,5–24,9^+^	–	–	0,962 0,396–2,338	1,558 0,464–5,234	1,074 0,382–3,016	0,823 0,188–3,600	0,451 0,124–1,641	0,681 0,132–3,502
**Tumorlokalisation Hypopharynx vs. Larynx**^**+**^	1,028 0,419–2,522	0,970 0,263–3580	4,036^***^ **2,267–7,184**	4,744^***^ **2,172–10,361**	3,380^***^ **1,830–6,245**	1,433 0,560–3,668	0,921 0,471–1,800	0,766 0,281–2,086
**T‑Stadium 3–4 vs. 1–2**^**+**^	–	–	1,418 0,757–2,665	2,593^*^ **1,091–6,163**	1,194 0,613–2,325	1,4	1,317 0,586–2,960	1,235 0,306–4,980
*Dysphagie gesamt*								
Dysphagie prätherapeutisch vs. keine Dysphagie^+^	–	–	–	–	–	–	0,354^*^ **0,126–0,995**	1,464 1,060–20,301
Dysphagie posttherapeutisch vs. keine Dysphagie^+^	–	–	–	–	–	–	0,652 0,172–2,463	2,455 0,506–11,905
Dysphagie prä- und posttherapeutisch vs. keine Dysphagie^+^	–	–	–	–	–	–	1,046 0,458–2,388	4,019 0,86–18,233
Dysphagie ja vs. nein^+^	0,258^*^ **0,084–0,790**	4,225 0,790–22,582	–	–	6,369^***^ **3,294–12,317**	12,509^***^ **4,166–37,559**	–	–
*Therapie*								
Primäre Op. mit adjuvanter RT/RCT vs. primäre Op. ohne RT/RCT^+^	–	–	–	–	1,979 0,703–5,573	3,484 0,688–17,651	0,587 0,170–2,026	0,469 0,60–3,673
Primäre RT/RCT vs. primäre Op. ohne RT/RCT^+^	–	–	–	–	4,125^**^ **1,530–11,121**	3,858 0,793–18,772	0,975 0,323–2,943	1,102 0,175–6,941
Andere Therapie/fehlend vs. primäre Op. ohne RT/RCT^+^	–	–	–	–	2,173 0,654–7,223	4,937 0,808–30,187	0,414 0,097–1,773	0,0983 0,108–8,910

^+^Referenzkategorie

Signifikanzniveau ^*^< 0,05 ^**^< 0,01 ^***^< 0,001

Leeres Feld (–): Es fand keine Untersuchung der Einflussvariable auf die Zielvariable statt

*BMI* Body-Mass-Index, *CCI* Charlson Comorbidity Index, *ECOG *Performance-Status der Eastern Cooperative Oncology Group, *KI *Konfidenzintervall, *m.* männlich, *OR* Odds Ratio, *RCT* Radiochemotherapie, *RT* Radiotherapie, *w. *weiblich

#### Untergewicht vor Therapie

Patienten mit ECOG 3–4 haben nach univariabler (*p* = 0,024/OR 4,444/95 %-KI: 1,212–16,302) wie multivariabler (*p* *=* 0,023/OR 10,832/95 %-KI: 1,397–83,997) Analyse ein signifikant höheres Risiko eines Untergewichts vor Therapiebeginn als Patienten mit ECOG 1–2. Ebenfalls signifikant in univariabler Analyse war die Reduktion des Risikos auf Untergewichtigkeit für Patienten mit prätherapeutischer Dysphagie (*p* = 0,018/OR 0,258/95 %-KI 0,084–0,790).

#### Dysphagie vor Therapie

Patienten mit Hypopharynxkarzinom hatten im Vergleich zu Larynxkarzinompatienten sowohl in univariabler (*p* < 0,001/OR 4,036/95 %-KI: 2,267–7,184) wie auch multivariabler Analyse (*p* < 0,001/OR 4,036/95 %-KI: 2,267–7,184) ein 4‑fach höheres Risiko einer Dysphagie vor Therapiebeginn. Patienten mit prätherapeutischem Untergewicht wiesen ein signifikant höheres Risiko einer prätherapeutischen Dysphagie auf (univariabel: *p* = 0,012/OR 4,330/95 %-KI: 1,372–13,670 multivariabel: *p* = 0,019/OR 7,558/95 %-KI 1,394–40,981). Die multivariable Analyse zeigte eine Verdopplung des Risikos für Dysphagie bei Tumorstadium T3–T4 (*p* < 0,031/OR 2,593/95 %-KI: 1,091–6,163), paradoxerweise eine Reduktion bei hohem Charlson Comorbidity Index (CCI, Score 3–4; *p* = 0,013/OR 0,229/95 %-KI: 0,071–0,737).

#### Dysphagie nach Therapie

Patienten, die vor Therapie eine Dysphagie aufwiesen, zeigten ein signifikant erhöhtes Risiko einer Dysphagie auch nach der Therapie, sowohl nach univariabler (*p* < 0,001/OR 6,369/95 %-KI: 3,294–12,317) als auch nach multivariabler (*p* < 0,001/OR 12,509/95 %-KI: 4,166–37,559) Analyse. In der univariablen Analyse zeigten Patienten mit einem Hypopharynxkarzinom ein 3,4-fach erhöhtes Risiko für eine posttherapeutische Dysphagie im Vergleich zu Larynxkarzinompatienten (*p* < 0,001/OR 3,380/95 %-KI: 1,830–6,245). Um das 4‑fache ist dieses Risiko für Patienten mit primärer Radiochemotherapie erhöht im Vergleich zu Patienten mit Operation (*p* = 0,005/OR 4,125/95 %-KI: 1,530–11,121). Gemäßigtes Übergewicht (BMI 25–29,9) zeigt sich in der multivariablen Analyse in Bezug auf Dysphagie eher protektiv im Vergleich zum Normalgewicht (*p* = 0,004/OR 0,228/95 %-KI: 0,084–0,623).

#### Pneumonie

In univariabler Analyse zeigten Patienten mit prätherapeutischer, jedoch ohne posttherapeutische Dysphagie mit *p* = 0,049 ein 0,35-fach geringeres Risiko einer Pneumonie im Vergleich zu Patienten ohne Schluckstörungen (95 %-KI: 0,126–0,995). Die multivariable Analyse ergab andererseits gerade nicht mehr signifikant ein 4‑fach erhöhtes Risiko (*p* = 0,071/95 %-KI: 0,86–18,233) einer Pneumonie bei Patienten mit prä- und posttherapeutischer Dysphagie.

### Überlebenszeitanalysen

**Abb. 2:**
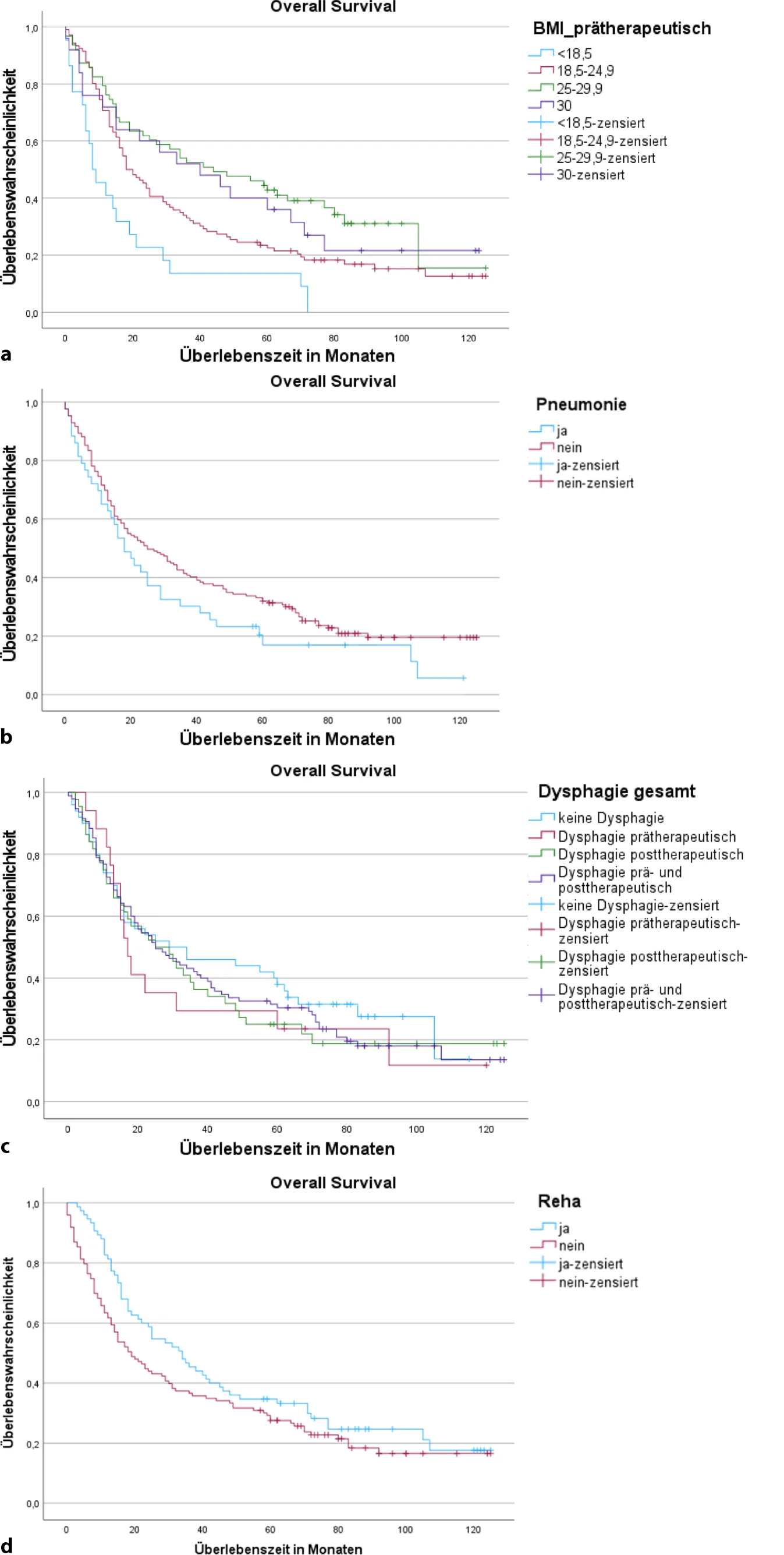
Kaplan-Meier-Kurven für das Gesamtüberleben („overall survival“, OS) in Abhängigkeit von **a** Body-Mass-Index (BMI), **b** Pneumonie, **c** Dysphagie, **d** Rehabilitation

Bei den Überlebenszeitanalysen in Abhängigkeit von dysphagie- und ernährungsabhängigen Einflussvariablen zeigte sich im Vergleich zu normalgewichtigen Patienten (Abb. 2a) ein signifikant schlechteres Überleben untergewichtiger (*p* = 0,003; Median 8 Überlebensmonate) und ein signifikant besseres Überleben moderat übergewichtiger (*p* = 0,017; Median 44 Überlebensmonate) Patienten.

In Bezug auf die anamnestisch erfasste Dysphagie (Abb. 2c) hingegen bestand kein signifikanter Unterschied zwischen den vier Kategorien. Für Patienten mit Pneumonie (Abb. 2b) ergab sich kein signifikanter Unterschied, jedoch ein tendenziell schlechteres Überleben (*p* = 0,109) mit einer medianen Überlebenszeit von 18 Monaten vs. 25 Monaten.

Auch Patienten, die nach onkologischer Therapie eine Rehabilitationseinrichtung (Abb. 2d) besuchten, zeigten kein signifikant besseres, sondern allein ein tendenziell besseres Überleben (*p* = 0,092) von im Median 34 Monaten im Vergleich zu 19 Monaten.

### Cox-Regression der Variablen in Bezug auf das Gesamtüberleben

#### Univariable Cox-Regression

Die Variablen ECOG, das Vorliegen von Fernmetastasen sowie der Noxenkonsum und der prätherapeutische BMI beeinflussten das Gesamtüberleben hoch signifikant (*p* < 0,001; linke Hälfte der Tab. 3): Das Risiko zu versterben stieg bei Patienten mit ECOG 3–4 im Vergleich zu ECOG 0–2 um das 3,4-fache (95 %-KI: 1,914–6,110), bei Fernmetastasen um das 2,3-fache (95 %-KI: 1,417–3,921), ebenso bei Zigaretten- (*p* = 0,015/HR 2,650/95 %-KI: 1,211–5,801) wie auch Alkoholabusus (*p* < 0,001/HR 7,183/95 %-KI: 2,512–20,537) im Vergleich zur Noxenabstinenz. Das signifikante Ergebnis aus der Gruppe mit alleinigem Alkoholkonsum ist aufgrund der geringen Fallzahl nur eingeschränkt aussagekräftig.

**Tab. 3:** Univariable und multivariable Cox-Regression

		Univariable Cox-Regression				Multivariable Cox-Regression			
Kategorie	Gruppe	*p*	HR	Unteres 95 %-KI	Oberes 95 %-KI	*p*	HR	Unteres 95 %-KI	Oberes 95 %-KI
Tumorentität	Larynx^+^	–	1	–	–	–	1	–	–
	Hypopharynx	0,110	1,287	0,945	1,754	0,337	1,254	0,790	1,988
Geschlecht	m.^+^	–	1	–	–	–	1	–	–
	w.	0,107	1,485	0,918	2,401	**0,07**	**2,383**	**1,275**	**4,456**
Diagnosealter (Jahre)	< 60^+^	0,107	1	–	–	0,480	1	–	–
	60–69,9	0,587	1,106	–	–	0,847	1,045	0,671	1,627
	70,0+	**0,040**	**1,478**	–	–	0,246	1,341	0,817	2,202
Primäre Therapie	Primäre Op. ohne postoperative Therapie^+^	**0,002**	**1**	–	–	0,125	1	–	–
	Primäre Op. mit postoperativer RT/RCT	0,786	0,918	0,495	1,703	0,839	0,928	0,451	1,910
	Primäre RT/RCT	0,079	1,658	0,942	2,916	0,157	1,663	0,822	3,365
	Keine Angaben	**0,020**	**2,148**	1,130	4,085	0,537	1,286	0,578	2,860
Dysphagie	Keine Dysphagie^+^	0,759	1	–	–	0,765	1	–	–
	Dysphagie vor Therapie	0,357	1,341	0,719	2,503	0,674	0,848	0,393	1,830
	Dysphagie nach Therapie	0,417	1,218	0,756	1,963	0,304	0,730	0,401	1,330
	Dysphagie vor oder nach Therapie	0,393	1,93	0,796	1,786	0,405	0,779	0,470	1,356
Tumorstadium	T1–2^+^	–	1	–	–	–	1	–	–
	T3–4	0,059	1,427	0,987	2,064	0,782	0,930	0,558	1,550
ECOG	0–2^+^	–	1	–	–	…	–	–	–
	3–4	**<0,001**	**3,421**	**1,915**	**6,110**	…	–	–	–
CCI nicht altersadjustiert	1–2^+^	0,416	1	–	–	0,824	1	–	–
	3–4	0,286	1,319	0,793	2,194	0,579	1,182	0,654	2,136
	≥5	0,185	1,425	0,844	2,405	0,544	1,215	0,647	2,284
Lymphknotenstatus positiv	Ja^+^	–	1	–	–	…	–	–	–
	Nein	0,175	0,782	0,548	1,116	…	–	–	–
Fernmetastasen	Ja	**<0,001**	**2,357**	**1,417**	**3,921**	0,881	1,063	0,477	2,371
	Nein^+^	–	1	–	–	–	1	–	–
Noxenkonsum	Keine^+^	**<0,001**	**1**	–	–	**0,038**	**1**	**–**	**–**
	Zigaretten	**0,015**	**2,650**	**1,211**	**5,801**	**0,034**	**2,748**	**1,079**	**6,997**
	Alkohol	**<0,001**	**7,183**	**2,512**	**20,537**	**0,008**	**14,300**	**2,033**	**…**
	Beides	0,142	1,661	0,844	3,268	0,104	1,980	0,869	4,512
BMI vor Therapie	<18,5	**0,004**	**2,015**	**1,258**	**3,230**	**0,033**	**2,025**	**1,058**	**3,875**
	18,5–24,9^+^	**<0,001**	**1**	**–**	**–**	**0,049**	**1**	**–**	**–**
	25–29,9	**0,018**	**0,634**	**0,435**	**0,924**	0,227	0,762	0,490	1,184
	≥30	0,236	0,735	0,442	1,223	0,935	0,975	0,534	1,782
Pneumonie	Ja	0,115	1,343	0,931	1,937	**0,041**	**1,569**	**1,018**	**2,420**
	Nein^+^	–	1	–	–	–	1	–	–
PEG	Ja^*^	**0,021**	**1,656**	**1,080**	**2,537**	…	–	–	–
	Nein^+^	–	1	–	–	–	–	–	–
Rehabilitation	Ja	0,097	0,758	0,546	1,051	0,437	0,850	0,563	1,282
	Nein^+^	–	1	–	–	–	1	–	–

^+^Referenzkategorie

Hervorgehoben in Fettdruck sind statistisch signifikante Ergebnisse

– Berechnung nicht möglich

*BMI* Body-Mass-Index, *CCI* Charlson Comorbidity Index, *ECOG *Performance-Status der Eastern Cooperative Oncology Group, *HR* Hazard Ratio, *KI *Konfidenzintervall, *m.* männlich, *PEG* perkutane endoskopische Gastrostomie, *RCT* Radiochemotherapie, *RT* Radiotherapie, *w. *weiblich

Das Sterblichkeitsrisiko stieg für Patienten mit Untergewicht im Vergleich zu Normalgewichtigen um das Doppelte an (*p* = 0,004/95 %-KI: 1,258–3,230), während leichtes Übergewicht dieses um etwa ein Drittel verminderte (*p* = 0,018/95 %-KI: 0,435–0,924).

Patienten mit PEG hatten ein höheres Sterberisiko als Patienten ohne Ernährungssonde (*p* = 0,021/HR 1,656/95 %-KI: 1,080–2,537).

Nicht signifikant war die Variable T‑Stadium mit *p* = 0,059 und einem 1,4-fach erhöhten Risiko (95 %-KI: 0,987–2,064) bei fortgeschrittenen Stadien. Auch zeigten Patienten mit stationärer Rehabilitation nur tendenziell ein niedrigeres Sterberisiko (0,76-fach, *p* = 0,097/95 %-KI: 0,546–1,051). Tendenziell stieg das Risiko bei Patienten mit diagnostizierter Pneumonie um das 1,3-fache (*p* = 0,115/95 %-KI: 0,931–1,937). Die über 70-jährigen Patienten zeigten ein um das 1,4-fache (*p* = 0,040) erhöhtes Risiko im Vergleich zu den unter 60-Jährigen.

#### Multivariable Cox-Regression

Die multivariable Analyse ergab für das Modell mit der besten Aussagekraft und den wenigsten Kollinearitäten (12 Kovariablen) insgesamt vier Einflussfaktoren (Tab. 3, rechts). Signifikanten Einfluss auf das Überleben, d. h. eine erhöhte Sterblichkeit, zeigten weibliches Geschlecht (*p* = 0,007, HR 2,383/95 %-KI: 1,275–4,456) und Noxenkonsum (*p* = 0,038); bei anamnestisch erhobenem Alkoholabusus ergab sich eine HR von 14,3 (*p* = 0,008), welche jedoch bei nur zwei Patienten nicht aussagekräftig erschien. Auch für die Subgruppen mit Zigaretten- (*p* = 0,034/HR 2,748/95 %-KI: 1,079–6,997) und kombiniertem Noxenkonsum (*p* = 0,104/HR 1,980/95 %-KI: 0,869–4,512) stieg das Risiko vergleichsweise an. Als wichtiger Einflussfaktor im Sinne der Fragestellung dieser Arbeit ließ sich jedoch eine Pneumonie ermitteln (*p* = 0,041); war diese während oder nach onkologischer Therapie dokumentiert, erhöhte sich das Sterberisiko um das 1,57-fache (95 %-KI: 1,018–2,420). Als weiterer, unabhängiger Risikofaktor erwies sich der prätherapeutische BMI, denn die Wahrscheinlichkeit zu versterben stieg für Untergewichtige um das 2,02-fache (*p* = 0,049, 95 %-KI: 1,058–3,875).

## Diskussion

Das Studienkollektiv zeigt eine typische Geschlechtsverteilung und Altersstruktur für Larynx- und Hypopharynxkarzinome, jedoch mit 35,9 % und 24,6 % ein ungewöhnlich niedriges 5‑Jahres-Überleben (gemäß Robert Koch-Institut [RKI]: 61 % und 47–63 %) [18]. Abweichend davon befanden sich jedoch im Studienkollektiv bereits 74 % der Larynxkarzinome und 71,5 % der Hypopharynxkarzinome im Stadium T3 oder T4, auch weil kleine Larynxkarzinome der Stimmlippen (T1/2) aufgrund mangelnder Schluckstörungen ausgeschlossen wurden. Ebenso war der Anteil multimorbider Patienten (altersadjustierter CCI-Score > 3) mit 80,6 % hoch gegenüber einer deutschen Vergleichspopulation [6], dies einhergehend mit einer erhöhten Einjahresmortalität. Das mittlere Überleben für Hypopharynxtumore zeigte sich hingegen mit 34 Monaten (Median: 18 Monate) im Vergleich zu Larynxtumoren mit 41 Monaten (Median: 30 Monate) wie erwartet niedriger [11].

Die multivariable COX-Regression identifizierte zwei unabhängige, ernährungs- und schluckabhängige Risikofaktoren mit signifikantem Einfluss auf das Gesamtüberleben: die Pneumonie und die Mangelernährung. Gleichwohl war die Erstellung eines Prognosemodells aufgrund wechselseitiger Einflüsse zwischen Ziel- und Einflussvariablen erschwert, weswegen uni- und multivariable Analysen zur Identifikation möglicher Mediatorvariablen durchgeführt wurden.

Pneumonien während oder nach onkologischer Therapie fanden sich in unserem Kollektiv mit mindestens 5‑jähriger Nachbeobachtungszeit bei 19 % der Patienten und erhöhten die Gesamtsterblichkeit um 57 %, ähnlich anderen register- und sekundärdatenbasierten Erhebungen [21]. Diese fanden bei einer 5‑Jahres-Prävalenz von 23,8 % eine um 42 % erhöhte Sterblichkeit [21]. Während sich dort Tumorlokalisationen in Hypo- und Nasopharynx, männliches Geschlecht, Alter, erhöhte Komorbidität und eine nichtchirurgische Therapie als Risikofaktoren einer Aspirationspneumonie identifizieren ließen, ähnlich wie auch an anderer Stelle [17], waren diese im vorliegenden kleineren Kollektiv nicht nachweisbar.

In unserer Studie traten Pneumonien 4‑fach häufiger (statistisch nicht signifikant) bei prä- wie posttherapeutisch dokumentierter Dysphagie auf. Dieser nichtsignifikante Zusammenhang könnte aufgrund von Schwächen der retrospektiven Datenerhebung, einer heterogenen, z. T. allein anamnestisch in der Krankenakte erfassten Dysphagie und Pneumonie begründet sein. Eine weitere Erklärung könnte auch darin liegen, dass auch nichttumorbedingte Komorbiditäten zu Pneumonien führen, wenngleich deutlich seltener [21]. Dies legt Aspiration und Pneumonien als Folge tumor-/therapiebedingter Dysphagie nahe. In der Tat ließen sich Hypopharynxtumore, höheres Tumorstadium, vorbestehende Mangelernährung bzw. das Geschlecht und eine primär nichtchirurgische Therapie als Risikofaktoren einer prä- bzw. posttherapeutischen Dysphagie identifizieren, an der 55 % bzw. 63,5 % der Patienten litten. Schwerer erklärbar scheint, warum höhere Komorbidität risikomindernd für eine prätherapeutische Dysphagie war. Eine mögliche Ursache könnte in der subjektiven Erhebung der Dysphagie liegen, welche stark von der Selbstwahrnehmung, Kognition und den Lebensumständen der Patienten abhängt. Dies war jedoch im retrospektiven Setting nicht mehr nachzuvollziehen.

Die genannten Einflussfaktoren lassen sich aber als Kriterien eines Risikoprofils benennen, gefährdete Patienten z. B. mittels eines instrumentellen Schluckscreenings zu identifizieren, welches Limitationen von Anamnese, PROM („patient-reported outcome measurements“) und klinischen Schluckscreenings [9, 10] vermeiden könnte. So können prä- und/oder (früh)rehabilitative Maßnahmen, wie instrumentell kontrollierte, kompensatorische Maßnahmen der funktionellen Dysphagietherapie, eingeleitet werden, um das Risiko einer Aspirationspneumonie zu minimieren [14].

Maßnahmen einer stationären Rehabilitation zeigten sich in dieser Studie allein in uni-, nicht aber multivariabler Analyse tendenziell protektiv. Erklärt wird dies möglicherweise durch das jüngere Alter, das Vorliegen von Dysphagien und Hypopharynxkarzinomen sowie einen Zustand nach Radio(chemo)therapie bei Patienten, die eine Rehabilitation in Anspruch nahmen.

Als zweiter ernährungs- und schluckabhängiger Risikofaktor des Gesamtüberlebens ließ sich mit 2‑fach erhöhter Sterblichkeit eine bereits vor onkologischer Therapie bestehende Mangelernährung identifizieren. Mangelernährung lag dabei mit einer Häufigkeit von nur 9,9 % deutlich unter anderen publizierten Daten von 25–65 %. [6, 20]. Ein Grund dafür könnte sein, dass die Mangelernährung, aufgrund der lückenhaften Datenlage, nur anhand des BMI festgestellt werden konnte, wichtige Faktoren zur Feststellung der Malnutrition, wie Serumalbumin, Gewichtsverlust in den letzten drei Monaten, konnten nicht suffizient erhoben werden [1]. Aufgrund des im zeitlichen Verlauf zu lückenhaft dokumentierten BMI konnten wir nur den BMI vor Therapiebeginn in die statistische Auswertung mit einbeziehen. Unklar ist der Einfluss im weiteren Verlauf der Therapie, zumal wir zeigen konnten, dass es eine tendenzielle Zunahme der untergewichtigen Patienten gibt (Abb. 1). Es ist zwingend erforderlich, das Gewicht/den BMI und auch andere Faktoren der Mangelernährung in die Dokumentation im Rahmen der Tumornachsorge aufzunehmen.

Als Einflussfaktor der Mangelernährung ließ sich die prätherapeutische Dysphagie in multivariabler Regression nur tendenziell erkennen, signifikant hingegen ein schlechterer ECOG. Während in univariabler Analyse trotz mehrfacher Überprüfung eine Dysphagie das Risiko prätherapeutischer Mangelernährung reduzierte. Patienten mit bestehender prätherapeutischer Mangelernährung hingegen haben nach multivariabler Analyse ein 7,5-fach erhöhtes Risiko für eine prätherapeutische Dysphagie. Dies könnte insofern verständlich sein, als dass Dysphagie, aufgrund mangelnder Differenzierung des Schweregrads in dieser Arbeit, zwar unregelmäßig zu Mangelernährung führt, Mangelernährung umgekehrt aber am ehesten aufgrund einer Dysphagie entsteht.

Weil Mangelernährung als Prädiktor früher Mortalität und früher Rezidive bekannt ist und als limitierender Faktor kurativer Therapien verstanden wird [2, 3], könnte der sehr frühzeitigen Behandlung nicht allein der Mangelernährung, sondern auch der zugrunde liegenden Dysphagie besondere Bedeutung zukommen. Ein Ernährungsscreening und -management vor Therapiebeginn wird bereits unabhängig vom BMI für alle onkologischen Patienten empfohlen [1, 10]. Um die Lebensqualität und auch das Überleben kachektischer Patienten zu verbessern, sind multimodale Therapieregime nötig [13].

Eine Wirkung potenziell protektiver Faktoren konnte in dieser Studie nicht belegt werden, weder stationäre Rehabilitation noch die PEG-Anlage. Im Gegenteil zeigte sich nach PEG-Anlage eine erhöhte Sterblichkeit, möglicherweise aufgrund eines prinzipiell fortgeschritteneren Tumorstadiums, vorbestehenden schlechteren Allgemeinzustands, vorbestehender Mangelernährung und einer Dysphagie. Die hier vorgelegten Daten berühren insofern nicht die Indikation einer präventiven PEG-Anlage [8]. Eine verbesserte Oralisierung hingegen könnte Mangelernährung nachhaltig bekämpfen, nutzbar z. B. im Rahmen einer prähabilitativen Schlucktherapie [20] und individuellen Ernährungsberatung, einer Fragestellung, der sich die aktuelle Proof-of-Concept-Studie PREHAPS des Gemeinsamen Bundesausschusses (G-BA) widmet [14]. Allein prätherapeutisches Übergewicht zeigte sich protektiv.

Daneben konnten die Daten der multivariablen Cox-Regression zeigen, dass ein Zigarettenkonsum das Risiko zu versterben 2,7-fach erhöht, für Alkoholkonsum sogar um das 17-fache. Diese Aussagen sind nur eingeschränkt interpretierbar, da in diesen Gruppen die Fallzahlen gering sind.

Eine deutliche Limitation der Studie ist das retrospektive Studiendesign. Zum einen konnten aufgrund fehlender Daten Teilbereiche nicht oder nur unzureichend ausgewertet werden, wie beispielsweise die Malnutrition nur anhand des BMI, zum anderen handelt es sich bei einigen Variablen teilweise nur um anamnestische Angaben, die mit einem gewissen Grad der Unsicherheit verbunden sind. Hierunter fallen z. B. auch die Dysphagie (nur in 64 % der Fälle liegt eine Schluckuntersuchung vor) und Angaben zum Noxenkonsum. Aus diesem Grund ist es in Zukunft nötig, Studien mit einem prospektiven Design und instrumenteller Schluckdiagnostik durchzuführen.

**Abb. 3:**
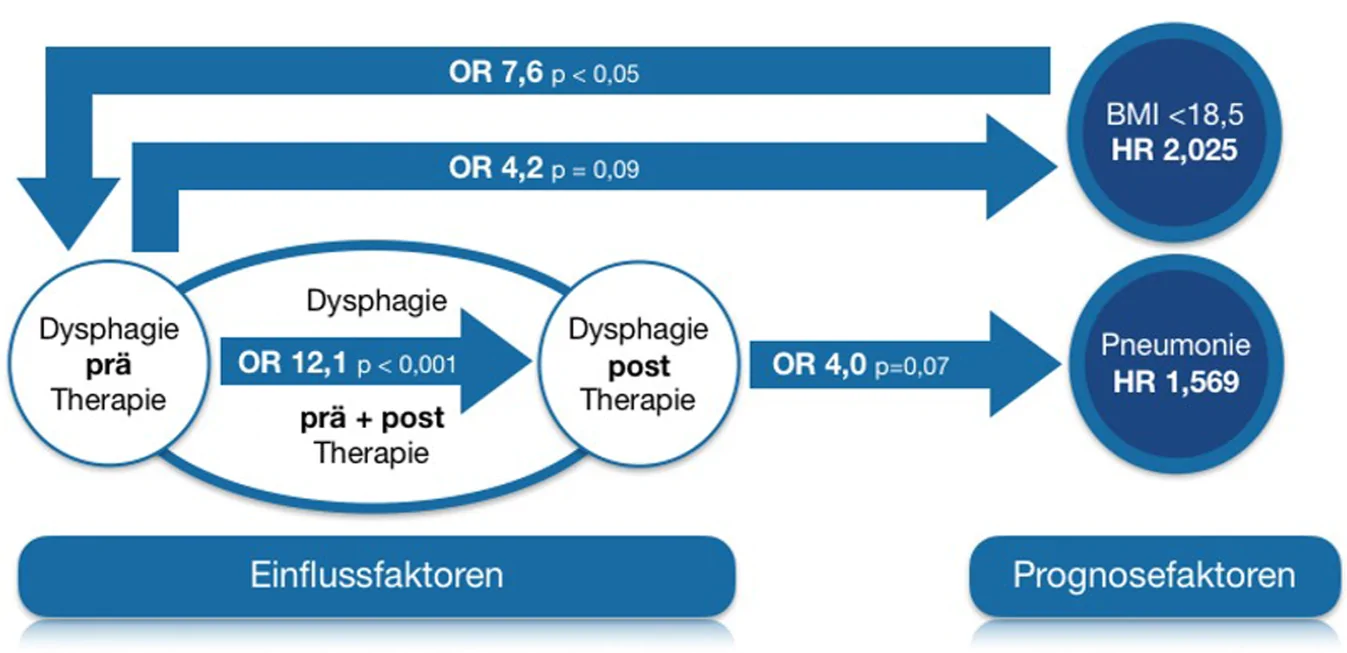
Prognosemodell der Einflussfaktoren auf das Gesamtüberleben („overall survival“, OS). *BMI* Body-Mass-Index, *HR* Hazard Ratio, *OR* Odds Ratio

Die mögliche zeitliche und kausale Darstellung der hier gefundenen, interventionsfähigen Einflussfaktoren einer prä- und/oder posttherapeutischen Dysphagie, Mangelernährung und Pneumonie und des damit verbundenen Sterblichkeitsrisikos zeigt Abb. 3.

## Fazit für die Praxis

- Pneumonien und Malnutrition, gemessen anhand des BMI, stellten an diesem retrospektiv untersuchten Kollektiv, mit überwiegend fortgeschrittenen Hypopharnx- und Larynkarzinomen, unabhängige Prognosefaktoren dar.
- Weitere Studien im deutschen Gesundheitssystem sind notwendig, um die Outcomebeurteilung prä-, früh- und stationärer Rehabilitationsmaßnahmen nach onkologischer Therapie jenseits allein subjektiver, patientenzentrierter Erfolgsmessung auf die mit Dysphagie verbundenen Folgen der Krankheitsbewältigung, Lebensqualität, Morbidität und Mortalität sowie der mit ihren Komplikationen verbundenen Kosten zu erweitern.
- Vor allem bereits vor Therapiebeginn bestehende Risiken der Mangelernährung und Dysphagie legen bereits zu diesem Zeitpunkt Maßnahmen im Sinne einer Prähabilitation nahe, während das Fortbestehen und aufgrund der Therapiemaßnahmen neu auftretende Störungen, frührehabilitative und klassische Maßnahmen stationärer Rehabilitation erfordern.

## Data Availability

Die erhobenen Datensätze können auf begründete Anfrage beim korrespondierenden Autor angefordert werden.

## References

[1] Capuano G, Gentile PC, Bianciardi F et al (2010) Prevalence and influence of malnutrition on quality of life and performance status in patients with locally advanced head and neck cancer before treatment. Support Care Cancer 18(4):433–437 10.1007/s00520-009-0681-8 19562384

[2] Chang P‑H, Wang C‑H, Huang J‑S et al (2012) Low body mass index at 3 months following adjuvant chemoradiation affects survival of postoperative locally advanced oral cavity cancer patients. Laryngoscope 122(10):2193–2198 10.1002/lary.23450 22886710

[3] Chang P‑H, Yeh K‑Y, Huang J‑S et al (2013) Pretreatment performance status and nutrition are associated with early mortality of locally advanced head and neck cancer patients undergoing concurrent chemoradiation. Eur Arch Otorhinolaryngol 270(6):1909–1915 10.1007/s00405-012-2290-2 23188165

[4] Chen S‑W, Yang S‑N, Liang J‑A et al (2010) The outcome and prognostic factors in patients with aspiration pneumonia during concurrent chemoradiotherapy for head and neck cancer. Eur J Cancer Care (engl) 19(5):631–635 10.1111/j.1365-2354.2009.01104.x 20109165

[5] Crowder SL, Douglas KG, Pepino YM et al (2018) Nutrition impact symptoms and associated outcomes in post-chemoradiotherapy head and neck cancer survivors: a systematic review. J Cancer Surviv 12(4):479–494 10.1007/s11764-018-0687-7 29556926

[6] Göllnitz I, Inhestern J, Wendt TG et al (2016) Role of comorbidity on outcome of head and neck cancer: a population-based study in Thuringia, Germany. Cancer Med 5(11):3260–3271 10.1002/cam4.882 PMC5119982 27726294

[7] Hoesseini A, van Leeuwen N, Offerman MPJ et al (2021) Predicting survival in head and neck cancer: External validation and update of the prognostic model OncologIQ in 2189 patients. Head Neck 43(8):2445–2456 10.1002/hed.26716 PMC8359959 33960553

[8] Institut für Qualität und Wirtschaftlichkeit im Gesundheitswesen (2023) Prophylaktische perkutane endoskopische Gastrostomiesonde; Evidenzbericht zur S3-Leitlinie Oropharyngeale Dysphagie infolge einer Kopf-Hals-Tumor-Erkrankung – Diagnostik und Therapie. IQWiG

[9] Institut für Qualität und Wirtschaftlichkeit im Gesundheitswesen (2023) Selbstevaluation des Schluckvermögens; Evidenzbericht zur S3-Leitlinie Oropharyngeale Dysphagie infolge einer Kopf-Hals-Tumor-Erkrankung – Diagnostik und Therapie. IQWiG

[10] Institut für Qualität und Wirtschaftlichkeit im Gesundheitswesen (2023) Triage-Tests im Rahmen der Dysphagiediagnostik; Evidenzbericht zur S3-Leitlinie Oropharyngeale Dysphagie infolge einer Kopf-Hals-Tumor-Erkrankung – Diagnostik und Therapie. IQWiG

[11] Jansen L, Moratin J, Behr C et al (2023) Epidemiologie von Kopf-Hals-Tumoren in Deutschland. Onkologie 29(1):5–11

[12] Johnson DE, Burtness B, Leemans CR et al (2020) Head and neck squamous cell carcinoma. Nat Rev Dis Primers 6(1):92 10.1038/s41572-020-00224-3 PMC7944998 33243986

[13] Kramer H, Alvarez PJC (2004) Prognostisch relevante Faktoren und Therapieergebnisse der stationären Dysphagiebehandlung. https://www.egms.de/static/en/meetings/dgpp2004/04dgpp47.shtml. Zugegriffen: 13. Mai 2024

[14] Kuenzel J, Duerr S, Vester S et al (2024) The effects of Phoniatric PREhabilitation in Head and Neck Cancer patients on Aspiration and Preservation of Swallowing (PREHAPS): study protocol of a monocentric prospective randomized interventional outcome-blinded trial. Trials 25(1):211 10.1186/s13063-024-08010-2 PMC10958950 38519961

[15] Loewen I, Jeffery CC, Rieger J et al (2021) Prehabilitation in head and neck cancer patients: a literature review. J Otolaryngol Head Neck Surg 50(1):2 10.1186/s40463-020-00486-7 PMC7789666 33407922

[16] Lubov J, Labbé M, Sioufi K et al (2021) Prognostic factors of head and neck cutaneous squamous cell carcinoma: a systematic review. J Otolaryngol Head Neck Surg 50(1):54 10.1186/s40463-021-00529-7 PMC8425113 34493343

[17] Purkey MT, Levine MS, Prendes B et al (2009) Predictors of aspiration pneumonia following radiotherapy for head and neck cancer. Ann Otol Rhinol Laryngol 118(11):811–816 19999368

[18] Robert Koch-Institut, Gesellschaft der epidemiologischen Krebsregister in Deutschland e. V. (2019) Krebs in Deutschland 2015/2016

[19] Seidl RO, Nusser-Müller-Busch R (2007) Schluckrehabilitation nach moderner Tumortherapie im Kopf-Hals-Bereich. Laryngorhinootologie 86(12):846–852 10.1055/s-2007-967028 17973200

[20] Vester S, Muhr A, Meier J et al (2023) Prehabilitation of dysphagia in the therapy of head and neck cancer—a systematic review of the literature and evidence evaluation. Front Oncol 13:1273430 10.3389/fonc.2023.1273430 PMC10766849 38188284

[21] Xu B, Boero IJ, Hwang L et al (2015) Aspiration pneumonia after concurrent chemoradiotherapy for head and neck cancer. Cancer 121(8):1303–1311 10.1002/cncr.29207 PMC4774546 25537836

[22] Zebralla V, Wichmann G, Pirlich M et al (2021) Dysphagia, voice problems, and pain in head and neck cancer patients. Eur Arch Otorhinolaryngol 278(10):3985–3994 10.1007/s00405-020-06584-6 PMC8382612 33452920

